# Arrhythmogenic triggers associated with Sudden Cardiac Death

**DOI:** 10.1080/19336950.2017.1388057

**Published:** 2017-11-13

**Authors:** Mena Abdelsayed, Colin H. Peters, Peter C. Ruben

**Affiliations:** Department of Biomedical Physiology and Kinesiology, Simon Fraser University, Burnaby, British Columbia, Canada

**Keywords:** Brugada syndrome, Long QT3 syndrome, voltage-gated sodium channels

Slightly more than two decades have passed since the discovery of the first *SCN5a* mutation, ΔKPQ, associated with inherited arrhythmias^1^. Ever since, a plethora of *SCN5a* mutations have been discovered and linked to syndromes such as Brugada Syndrome type 1 (BrS1) and Long-QT syndrome type 3 (LQT3). Interestingly, some sodium channel mutants may cause a mixed syndrome of both BrS1 and LQT3, with the most common being E1784K^2^. Although E1784K may express a phenotypic overlap of both BrS1 and LQT3, the LQT3 phenotype is more common, especially in the Okinawa islands, where over 80% of LQT genotype positive children carry this mutation^3^.

*SCN5a* encodes the cardiac sodium channel, Na_V_1.5, which passes the transient inward sodium current underlying depolarization in ventricular myocytes. The E1784K mutant occurs in the Na_V_1.5 C-terminus, near a paired EF-hand like domain. The C-terminus in sodium channels is an important modulator of channel activation, fast inactivation, and slow inactivation. This modulation is in part due to a calcium-calmodulin dependent interaction between the C-terminus and the Domain III-IV linker, the sodium channel fast inactivation “particle”^4^. The charge-reversal mutant, E1784K, may disrupt the overall integrity of the C-terminus by disrupting the electrostatic forces connecting the EF-hand domain and the IQ motif. These interactions in the C-terminus are crucial for proper inactivation^4^. Structural and biophysical studies show that a disassembly in the C-terminus correlates with perturbed inactivation voltage-dependence and the presence of persistent sodium currents^4^. The E1784K channotype shares common biophysical perturbations with a number of other C-terminal LQT3 and BrS1 mutants, hyperpolarizing the voltage-dependence of steady-state fast inactivation (SSFI), depolarizing the voltage-dependence of activation (GV), and increasing the late sodium current^2^.

To better understand the pathophysiology of E1784K, we studied several physiological triggers associated with exercise that are known to unmask arrhythmias. We have shown that increases in body temperature increase the fraction of non-inactivating channels passing late sodium current in E1784K^5^. We also showed that E1784K is sensitive to changes in extracellular protons^6^. Decreases in extracellular pH greatly decreased peak sodium currents and increased the fraction of non-inactivating E1784K sodium channels. Action potential simulations based on the ten Tüsscher model of the ventricular action potential suggested that E1784K would cause a large transmural dispersion of repolarization with increases in temperature or decreases in extracellular pH. Our pH results with WT and E1784K channels allowed us to generate a novel model, the Peters-Ruben model of sodium channel gating^6^. This model confirms the E1784K channotype arises from defects in fast inactivation, and predicts the mutant disrupts interactions between the C-terminus and the voltage sensor in Domain 4, translocation of which has been shown to be linked to fast inactivation^7^.

We recently reported the effects of increasing cytosolic calcium on E1784K and other sodium channel mutants^8^. Calcium-sensitivity is differentially affected by *SCN5a* mutations. Mutants in channel regions regulating inactivation which enhance non-inactivating sodium current include ΔKPQ, E1784K, 1795insD, and Q1909R. We showed that cytosolic calcium blocks late sodium current in all these mutants except in E1784K^8^. We proposed that E1784K completely abolishes the regulatory effects mediated by calcium-calmodulin on the fast inactivation particle and the C-terminus, whereas the other mutants do not disrupt this regulatory mechanism. Elevations in cytosolic calcium also restores the voltage-dependence of steady-state slow inactivation to the wild-type form in all mutants except E1784K, which was depolarized by cytosolic calcium, thereby increasing channel availability. To further understand the gain-of-function observed in E1784K under conditions consistent with high heart rates, we simulated our results using the O'Hara Rudy Model. Simulated action potentials show that the highly temperature and calcium-sensitive E1784K mutant prolongs the AP and produces alternans at high frequencies.

In conclusion, E1784K carriers may display Long-QT or BrS1 phenotypes, or both; however, these diagnostic factors are not always sufficient to induce high-risk arrhythmias in those patients. Rather, a perfect storm of events is met when external triggers, such as those associated with exercise, perturb channel behaviour and alter phenotypic expressivity. Also clear from our results is that not all *SCN5a* mutations are subject to the same arrhythmogenic triggers. In other words, what is dangerous for one cardiac patient might be beneficial for another. Our findings support the case for detailed biophysical assessment of individual channel mutants and personalized, precision medicine, including genotyping, for cardiac patients who present with evidence of inherited arrhythmias.
